# Profile, treatment patterns, and influencing factors of anthracycline use in breast cancer patients in China: A nation‐wide multicenter study

**DOI:** 10.1002/cam4.4215

**Published:** 2021-09-02

**Authors:** Fengzhu Guo, Zongbi Yi, Wenna Wang, Yiqun Han, Pei Yu, Su Zhang, Quchang Ouyang, Min Yan, Xiaojia Wang, Xichun Hu, Zefei Jiang, Tao Huang, Zhongsheng Tong, Shusen Wang, Yongmei Yin, Hui Li, Runxiang Yang, Huawei Yang, Yuee Teng, Tao Sun, Li Cai, Hongyuan Li, Xi Chen, Jianjun He, Xinlan Liu, Shune Yang, Jinhu Fan, Youlin Qiao, Jiayu Wang, Binghe Xu

**Affiliations:** ^1^ Department of Medical Oncology National Cancer Center/National Clinical Research Center for Cancer/Cancer Hospital Chinese Academy of Medical Sciences and Peking Union Medical College Beijing China; ^2^ State Key Laboratory of Molecular Oncology National Cancer Center/National Clinical Research Center for Cancer/Cancer Hospital Chinese Academy of Medical Sciences and Peking Union Medical College Beijing China; ^3^ Department of Cancer Epidemiology National Cancer Center/National Clinical Research Center for Cancer/Cancer Hospital Chinese Academy of Medical Sciences and Peking Union Medical College Beijing China; ^4^ Department of Breast Cancer Medical Oncology Hunan Cancer Hospital Changsha China; ^5^ Department of Breast Disease Henan Breast Cancer Center The affiliated Cancer Hospital of Zhengzhou University & Henan Cancer Hospital Zhengzhou China; ^6^ Department of Medical Oncology Zhejiang Cancer Hospital Hangzhou China; ^7^ Department of Medical Oncology Fudan University Shanghai Cancer Center Shanghai China; ^8^ Department of Breast Cancer The Fifth Medical Centre of Chinese PLA General Hospital Beijing China; ^9^ Department of Breast and Thyroid Surgery Union Hospital Tongji Medical College Huazhong University of Science and Technology Wuhan China; ^10^ Department of Breast Oncology Key Laboratory of Breast Cancer Prevention and Therapy National Clinical Research Center for Cancer Tianjin Medical University Cancer Institute and Hospital Tianjin China; ^11^ Department of Medical Oncology State Key Laboratory of Oncology in South China Sun Yat‐sen University Cancer Center Guangzhou China; ^12^ Department of Medical Oncology The First Affiliated Hospital of Nanjing Medical University Nanjing China; ^13^ Department of Breast Surgery Sichuan Province Tumor Hospital Chengdu Sichuan China; ^14^ Department of Medical Oncology Yunnan Cancer Hospital Kunming Medical University Kunming China; ^15^ Department of Breast Surgery Cancer Hospital Guangxi Medical University Guangxi China; ^16^ Departments of Medical Oncology and Thoracic Surgery The First Hospital of China Medical University Shenyang China; ^17^ Department of Medical Oncology Cancer Hospital of China Medical University Liaoning Cancer Hospital and Institute Key Laboratory of Liaoning Breast Cancer Research Shenyang China; ^18^ The 4th Department of Internal Medical Oncology Harbin Medical University Cancer Hospital Harbin China; ^19^ Department of the Endocrine and Breast Surgery The First Affiliated Hospital of Chongqing Medical University Chongqing Medical University Chongqing China; ^20^ Department of Medicine Oncology 900 Hospital of the Joint Logistics Team Fuzhou China; ^21^ Department of Breast Surgery The First Affiliated Hospital of Xi’an Jiaotong University Xi’an China; ^22^ Department of Oncology General Hospital of Ningxia Medical University Yinchuan Ningxia China; ^23^ Department of Breast Cancer and Lymphoma Affiliated Tumor Hospital of Xinjiang Medical University Urumqi China

**Keywords:** anthracycline, breast cancer, chemotherapy, epidemiological study, nation‐wide

## Abstract

**Background:**

Anthracycline‐based chemotherapy (ABC) is one of the standard therapies against breast cancer. However, few guidelines are currently available to optimize the use of ABC. Therefore, the present analysis aimed at determining the profile and treatment patterns of ABC and the association of clinicopathological characteristics with ABC selection.

**Methods:**

We retrospectively analyzed the data of a nation‐wide multicenter epidemiological study, which collected the medical records of breast cancer patients receiving chemotherapy in different settings from seven geographic regions in China (NCT03047889).

**Results:**

In total, 3393 patients were included, with 2917 treated with ABC. Among them, 553 (89.8%), 2165 (81.7%), and 814 (25.7%) were subjected to ABC as neoadjuvant, adjuvant, and advanced chemotherapy, respectively. The most frequently used regimens were anthracycline‐taxane‐based combinations for neo‐ and adjuvant chemotherapy, along with taxanes and oral fluorouracils for the palliative stages. In the overall cohort, patients aged < 40 or 40‐65 (*p* < 0.001), in premenopause (*p* < 0.001), without comorbidities (*p* = 0.016), with invasive ductal carcinoma (*p*= 0.001), high lymph node involvement (*p* < 0.001), in the pTNM stage II, III, or IV versus stage I (*p* < 0.001), subjected to mastectomy (*p* < 0.001) or subjected to sentinel lymph node biopsy combined with axillary lymph node dissection (*p* = 0.044), or with a decreased disease‐free survival (*p* < 0.001) were more likely to be recommended to ABC.

**Conclusion:**

Taken together, ABC remained the mainstay of breast cancer treatment, especially in neo and adjuvant therapy. ABC was mainly used as a combination therapy, and the correlation between influencing factors and ABC choice varied during different settings, indicating the preference and different perspectives of medication considered by medical oncologists regarding the use ABC in China.

## Graphical abstract

The nation‐wide study revealed that anthracycline‐based chemotherapy (ABC) remained the mainstay of breast cancer treatment, especially in neo and adjuvant therapy, and was predominantly used as a combination therapy. The correlation between clinicopathological characteristics and ABC choice varied at different settings, suggesting the preference and different perspectives of medication regarding the use ABC in China. The real‐world data contribute to guide the selection of the appropriate treatment and maximize the benefit of chemotherapy for patients.

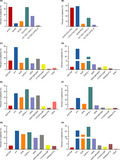

## 1 INTRODUCTION

Breast cancer has become the most common malignancy worldwide (11.7% of the total new cases) surpassing lung cancer, according to the latest estimates on the global cancer burden in 2020 released by the International Agency for Research on Cancer (IARC).^1^ Breast cancer is also the most commonly diagnosed cancer among Chinese women, with new cases accounting for approximately 20% of the total new cases of cancer in 2020, ranking fourth in terms of cancer mortality, accounting for approximately one in ten cancer deaths.^1^ Although the noteworthy progress of intensive endocrine therapy and effective targeted therapy, cytotoxic chemotherapy still plays a dominant role in the clinical treatment of breast cancer. In the context of precision medicine, breast cancer has entered the era of classified treatment, and chemotherapeutic strategies need to be individualized.^2^ Therefore, the application of chemotherapy in routine clinical practice is still the object of intense research.

As a class of antineoplastics, anthracyclines (such as doxorubicin and epirubicin) substantially improved the disease‐specific survival and are recognized as a standard therapy to combat breast cancer as demonstrated by the results of several clinical trials.^3^, ^4^, ^5^, ^6^, ^7^ Currently, regimens that include doxorubicin are listed in The National Comprehensive Cancer Network guidelines as an alternative option in all risk levels and stages of breast cancer except for the lowest risk and early‐stage breast cancer.^3^ However, the use of other anticancer agents has led to the study of a greater number of combinations of chemotherapeutic drugs in specific patients, and consequently anthracycline‐based chemotherapy (ABC) has been in decline for decades.^8^, ^9^ Additionally, anthracyclines exert remarkable adverse effects and among them, cardiotoxicity is the main one. Mounting evidence indicates that anthracyclines induce cardiotoxicity through topoisomerase‐2β inhibition together with oxidative stress mediated by reactive oxygen species generation.^10^ It also increases the risk of treatment‐related acute myeloid leukemia and myelodysplastic syndrome.^11^ Thus, it is essential to further clarify the factors involved in side effects and identify the population who may be prone to receive ABC.

Therefore, this retrospective clinical analysis enrolled 3387 patients with advanced breast cancer from seven geographic areas who received chemotherapy as neoadjuvant, adjuvant, or advanced palliative treatment, to explore the current overview and therapeutic patterns of anthracycline use, and determine the clinicopathological factors influencing the selection of ABC in different settings in China. The initial recurrence after adjuvant chemotherapy was also discussed, optimizing the clinical ABC use and patient management.

## 2 MATERIALS AND METHODS

### 2.1 Study design and data collection

A retrospective observational analysis was performed using the data of a hospital‐based multicenter epidemiological survey launched in 2015 (clinical trials.gov identifier: NCT03047889). The program collected medical information of advanced breast cancer patients between January 1, 2012, and December 31, 2014, involving 21 hospitals in seven different geographic regions in China, representing distinctive breast cancer burden levels. The National Cancer Center/National Clinical Research Center for Cancer/Cancer Hospital, Chinese Academy of Medical Sciences and Peking Union Medical College was the leading center for coordinating the overall research. Critical data included demographic information, medical history, disease characteristics, clinical management, and follow‐up. The designed case report form was used to obtain all the aforementioned information from the electronic medical record systems by trained oncologists. The eligibility criteria were the following: advanced breast cancer patients who were first treated in each collaborative hospital from January 1, 2012, to December 31, 2014, including patients with recurrent and metastatic breast cancer diagnosed before 2012. The exclusion criterion was represented by the lack of availability of the medical records.

An enrollment scheme in the form of alternating pre‐specified months for inpatient admission from year to year was adopted to avoid selection bias. Since aside from the Spring Festival, hospitalizations are similar during other months of the year, each hospital was assigned random numbers of month to make the population more representative and the program more feasible. In each selected month, additional patients from adjacent months were reviewed if fewer inpatients were included than the intended number until the total for the year reached the target quantity.

In this study, the demographic features and clinicopathological characteristics of the patients such as age, menopausal status, body mass index, comorbidity, family history of breast cancer, grade, stage, molecular subtype, and therapeutic approaches were evaluated. The primary endpoint was to investigate the current profile and treatment patterns of anthracycline use in real‐world clinical practice in China. The secondary endpoint was to determine factors associated with anthracycline selection.

### 2.2 Patient selection

**FIGURE 1:**
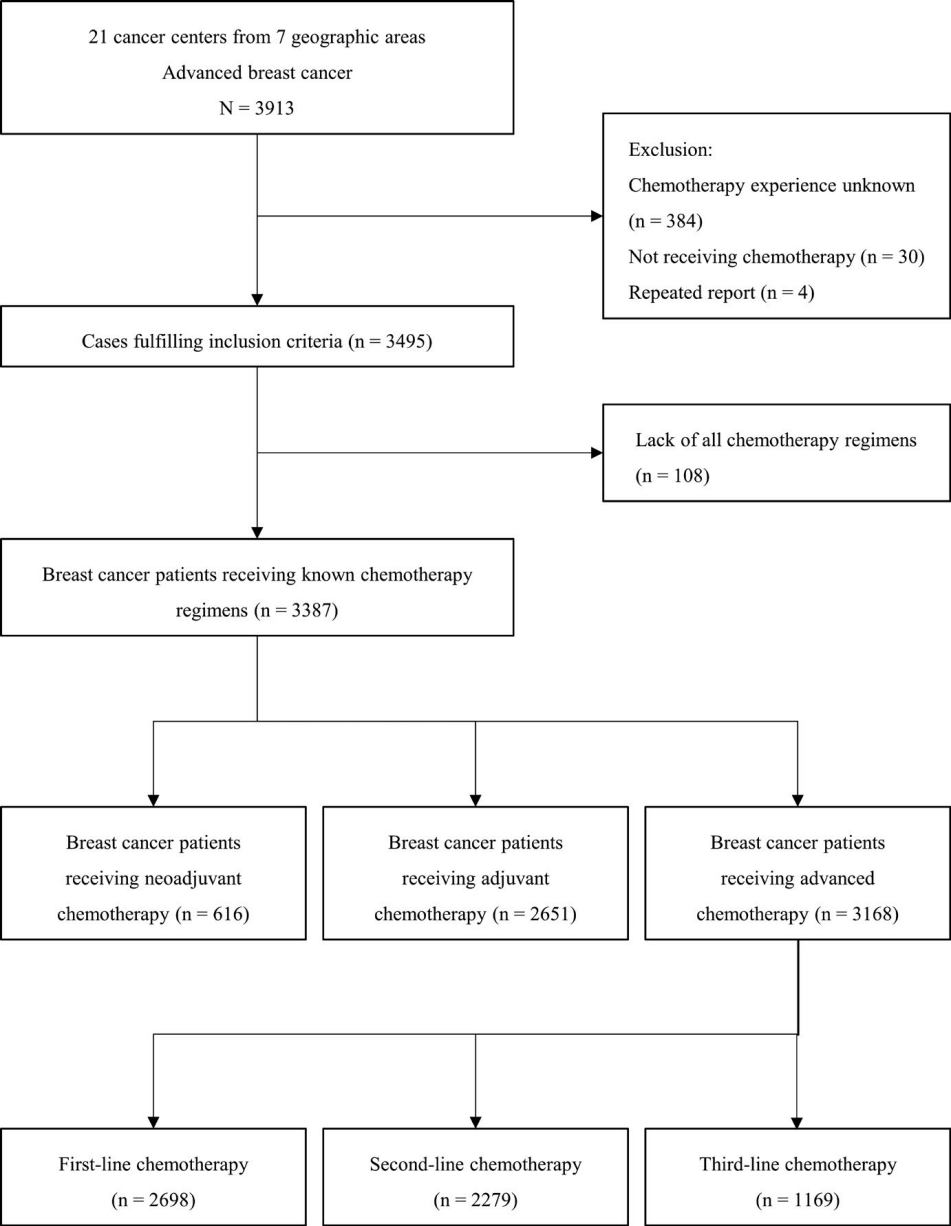
Flowchart of the study and the inclusion of patients subjected to chemotherapy at neoadjuvant, adjuvant, and advanced settings

Our analysis process is summarized in Figure 1. A total of 3913 patients with advanced breast cancer were originally enrolled in this epidemiological program. Among them, 3495 fulfilled the inclusion criteria while 384 were excluded because it was unknown whether they received chemotherapy, 30 because of not being subjected to chemotherapy, and four because of repeated reports. Among the remaining patients, 3387 receiving available chemotherapy regimens were identified after the exclusion of 108 due to lack of all chemotherapeutic data. Finally, the data of 616 patients treated with neoadjuvant chemotherapy, 2651 patients subjected to adjuvant chemotherapy, and 3168 patients treated with palliative chemotherapy were analyzed, of which 2698, 2279, and 1169 received first‐, second‐, and third‐line chemotherapy, respectively. In our research, when patients received certain agents/regimens, it was specified whether they used specific agents/regimens alone or in combination with other therapies. Some patients received chemotherapy at more than one setting.

### 2.3 Statistical analysis

Categorical variables and continuous variables not normally distributed were presented as frequency plus percentage and median plus interquartile range. Differences in clinicopathological features between two groups were examined by the chi‐square test or Fisher exact probability test and Wilcoxon rank‐sum test for categorical variables and rank variables, respectively. All statistical tests were two‐sided, and a value of *p* < 0.05 was considered statistically significant. Statistical analysis and graph drawing were performed using SPSS Statistics 23.0 (IBM Corporation) and GraphPad Prism 7.0 (GraphPad Software).

## 3 RESULTS

### 3.1 Demographic and clinicopathological characteristics

The entire cohort was composed of 2917 (86.0%) patients who used ABC in different treatment stages as opposed to 476 (14.0%) patients who had never been treated with any anthracyclines. The main clinicopathological characteristics of the enrolled patients are listed in Table 1. In general, patients aged <40 or 40–65 versus ≥65 years (88.1% for <40 years, 86.2% for 40–65 years, 65.3% for ≥65 years, *p* < 0.001), in premenopause versus postmenopause (88.4% vs. 83.1%, *p* < 0.001), with lymph node stage 1, 2 or 3 versus stage 0 (80.4% for stage 0, 88.0% for stage 1, 90.2% for stage 2, 90.9% for stage 3, *p* < 0.001), in the pTNM stage II, III, or IV versus stage I (80.9% for stage I, 88.4% for stage II, 91.0% for stage III, 84.3% for stage IV, *p* < 0.001), with invasive ductal carcinoma (IDC) versus non‐IDC (88.5% for IDC, 83.4% for non‐IDC, *p =* 0.001), subjected to mastectomy versus lumpectomy (87.2% vs. 85.4%, *p* < 0.001), or with a decreased disease‐free survival (DFS, 28.0 m, IQR 15.0–51.0, *p* < 0.001) were greatly prone to anthracycline‐contained regimens. Furthermore, our results revealed that ABC was more recommended in patients without systemic comorbidities than in those with comorbidities (87.1% vs. 83.4%, *p =* 0.016), and in those subjected to sentinel lymph node biopsy (SLNB) combined with axillary lymph node dissection (ALND) than in those with other lymph node examination (90.1% for SLNB +ALND, *p =* 0.044, Table 1).

**TABLE 1:** Overall clinicopathological characteristics and treatment regimens of the enrolled patients

Characteristics	All subjects, No.	Anthracycline‐based chemotherapy^a^, No. (%)	Anthracycline‐free chemotherapy^b^, No. (%)	*p*‐value
Patients (*n*)	3393	2917 (86.0)	476 (14.0)	
Age at diagnosis (years)				**<0.001**
<40	939	827 (88.1)	112 (11.9)	
40–65	2336	2013 (86.2)	323 (13.8)	
≥65	118	77 (65.3)	41 (34.7)	
Menopausal status				**<0.001**
Premenopausal	1566	1384 (88.4)	182 (11.6)	
Postmenopausal	1582	1315 (83.1)	267 (16.9)	
Unknown	245	218	27	
BMI				0.453
Normal (<24)	1643	1408 (85.7)	235 (14.3)	
Overweight (≥24)	1385	1200 (86.6)	185 (13.4)	
Unknown	365	309	56	
Comorbidity				**0.016**
Yes	639	533 (83.4)	106 (16.6)	
No	2574	2241 (87.1)	333 (12.9)	
Unknown	180	143	37	
Family history of breast cancer				0.676
Yes	147	128 (87.1)	19 (12.9)	
No	3059	2626 (85.8)	433 (14.2)	
Unknown	187	163	24	
Tumor grade (IDC)				0.286
I+II	981	875 (89.2)	106 (10.8)	
III	571	519 (90.9)	52 (9.1)	
NA or Unknown	1841	1523	318	
Tumor size stage				0.475
1	613	536 (87.4)	77 (12.6)	
2	1291	1147 (88.8)	144 (11.2)	
3	346	307 (88.7)	39 (11.3)	
4	13	10 (76.9)	3 (23.1)	
Unknown	1130	917	213	
Lymph node stage				**<0.001**
0	1323	1064 (80.4)	259 (19.6)	
1	823	724 (88.0)	99 (12.0)	
2	676	610 (90.2)	66 (9.8)	
3	571	519 (90.9)	52 (9.1)	
pTNM stage				**<0.001**
I	251	203 (80.9)	48 (19.1)	
II	955	844 (88.4)	111 (11.6)	
III	1056	961 (91.0)	95 (9.0)	
IV	261	220 (84.3)	41 (15.7)	
Unknown	870	689	181	
Histological subtype				**0.001**
IDC	2539	2246 (88.5)	293 (11.5)	
non‐IDC	574	479 (83.4)	95 (16.6)	
Unknown	280	192	88	
Breast surgery				**<0.001**
Mastectomy	2745	2394 (87.2)	351 (12.8)	
Lumpectomy	268	229 (85.4)	39 (14.6)	
No surgery	285	217 (76.1)	68 (23.9)	
Other	62	55 (88.7)	7 (11.3)	
Unknown	33	22	11	
Lymph node examination				**0.044**
SLNB	41	34 (82.9)	7 (17.1)	
ALND	2282	2034 (89.1)	248 (10.9)	
SLNB+ALND	71	64 (90.1)	7 (9.9)	
No surgery	128	106 (82.8)	22 (17.2)	
Other	10	7 (70.0)	3 (30.0)	
Unknown	861	672	189	
ER status				0.728
Positive	1648	1442 (87.5)	206 (12.5)	
Negative	1177	1035 (87.9)	142 (12.1)	
Unknown	568	440	128	
PR status				0.924
Positive	1471	1289 (87.6)	182 (12.4)	
Negative	1361	1191 (87.5)	170 (12.5)	
Unknown	561	437	124	
HER2 status				0.625
Positive	1016	892 (87.8)	124 (12.2)	
Negative	1633	1444 (88.4)	189 (11.6)	
Unknown	744	581	163	
Ki−67 (%)				0.714
<40	835	752 (90.1)	83 (9.9)	
≥40	492	440 (89.4)	52 (10.6)	
Unknown	2066	1725	341	
DFS (months)				**<0.001**
Median (IQR)		28.0 (15.0–51.0)	38.0 (18.0–76.75)	

Significant *p*‐values are indicated in bold.

Abbreviations: ALND, axillary lymph node dissection; BMI, body mass index; DFS, disease‐free survival; ER, estrogen receptor; HER2, human epidermal growth factor receptor 2; IDC, invasive ductal carcinoma; IQR, interquartile range; NA, not applicable; PR, progesterone receptor; pTNM, pathology TNM; SLNB, sentinel lymph node biopsy.

^a^ Patients who received anthracyclines in neoadjuvant, adjuvant, or advanced chemotherapy.

^b^ Patients who did not receive anthracyclines in neoadjuvant, adjuvant, or advanced chemotherapy.

### 3.2 Profile and treatment patterns of anthracycline usage at different chemotherapeutic settings

**FIGURE 2:**
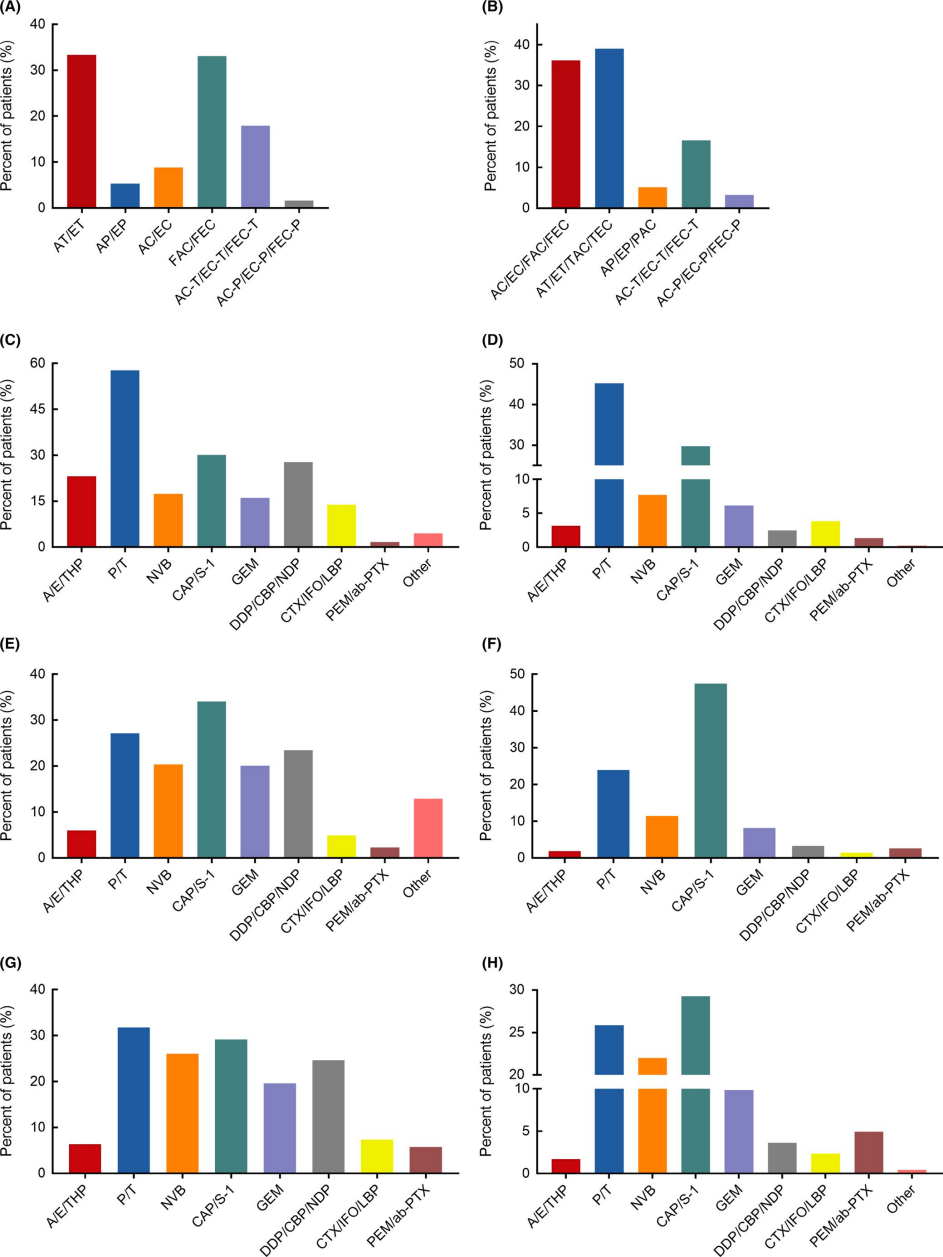
Anthracycline usage at different chemotherapeutic settings. (A) Regimens used as neoadjuvant chemotherapy. (B) Regimens used as adjuvant chemotherapy. (C‐H) Cytotoxic agents used as advanced first‐line chemotherapy (C) and monotherapy (D), second‐line chemotherapy (E) and monotherapy (F), and third‐line chemotherapy (G) and monotherapy (H). Abbreviations: A, doxorubicin; T, docetaxel; E, epirubicin; P, paclitaxel; C/CTX, cyclophosphamide; F, fluorouracil; THP, pirarubicin; NVB, vinorelbine; CAP, capecitabine; GEM, gemcitabine; DDP, cisplatin; CBP, carboplatin; NDP, nedaplatin; IFO, ifosfamide; LBP, lobaplatin; PEM, pemetrexed; ab‐PTX, albumin‐bound paclitaxel

Among the patients subjected to neoadjuvant chemotherapy, 553 (89.8%) received ABC. Among these (Figure 2A), doxorubicin plus docetaxel (AT)/epirubicin plus docetaxel (ET) was the most used protocol (33.3%), followed by fluorouracil plus doxorubicin and cyclophosphamide (FAC)/fluorouracil plus epirubicin and cyclophosphamide (FEC) (33.1%) and doxorubicin plus cyclophosphamide followed by docetaxel (AC‐T)/epirubicin plus cyclophosphamide followed by docetaxel (EC‐T)/fluorouracil, epirubicin plus cyclophosphamide followed by docetaxel (FEC‐T) (17.9%).

Among the patients subjected to adjuvant treatment, approximately 81.7% received ABC. The protocol consisting of AT/ET/docetaxel plus doxorubicin and cyclophosphamide (TAC)/docetaxel plus epirubicin and cyclophosphamide (TEC) represented the 39.0% of ABC. Furthermore, AC‐T/EC‐T/FEC‐T represented the 16.6% and was ranked as the third most frequently used regimens following doxorubicin plus cyclophosphamide (AC)/epirubicin plus cyclophosphamide (EC)/FAC/FEC, which represented the 36.1% (Figure 2B).

A large proportion of patients (76.9%) in the palliative treatment group received anthracycline‐free chemotherapy (AFC) at the first‐line setting. As regard the chemotherapeutic agent used (Figure 2C), more than half of the patients (57.7%) were treated with paclitaxel (P)/docetaxel (T), approximately 30.1% were treated with capecitabine (CAP)/S‐1, and 27.7% were treated with cisplatin (DDP)/carboplatin (CBP)/nedaplatin (NDP). Anthracyclines, including doxorubicin (A)/epirubicin (E)/pirarubicin (THP), represented the 26.5% among all chemotherapeutic agents, of which over 98% were anthracycline‐containing combination, and A/E/THP plus P/T was the most used protocol. A total of 440 (16.3%) received monotherapy, with P/T still the most common option, accounting for nearly half of all monotherapies (45.2%), and anthracyclines constituting only the 3.2% (Figure 2D). During the second‐line setting, CAP/S‐1 (34.1%), P/T (27.1%), and DDP/CBP/NDP (23.5%) were the top three most frequently used selections, and anthracyclines made up for the 6.0% (Figure 2E). Among these patients, combination therapy was the main form of ABC (91.2%), and A/E/THP plus P/T regimen was the predominant one. A total of 648 (41.2%) were treated with single‐agent chemotherapy, with CAP/S‐1 being the most common used drug, and anthracyclines representing the 1.9% of the total (Figure 2F). As regard the third‐line treatment, anthracyclines accounted for the 6.3% of all chemotherapies, with P/T, CAP/S‐1 and vinorelbine as the top three used agents (Figure 2G). A total of 88.9% of patients underwent ABC as the selected combined treatment, mostly A/E/THP plus P/T regimen. Among 468 patients who received monotherapy, CAP/S‐1 still ranked first, and anthracyclines constituted the 1.7% (Figure 2H).

### 3.3 Baseline factors associated with the selection of ABC as neoadjuvant chemotherapy

The data of 616 patients who received neoadjuvant chemotherapy were analyzed and among them, 553 (89.8%) were treated with ABC and 63 (10.2%) were treated with AFC (Table 2). ABC was administered more substantially to patients with a large tumor size (stage 3, 95.7%, *p =* 0.021) or cTNM stage III disease (94.4%, *p =* 0.009). Furthermore, patients with tumor grade III or with IDC nearly received significantly more ABC compared with patients with tumor grade I or II (95.3% vs. 83.5%, *p =* 0.056) and those with non‐IDC (91.2% vs. 85.5%, *p =* 0.056), respectively. No association between other baseline characteristics and chemotherapeutic protocols was observed during the neoadjuvant stage.

**TABLE 2:** Analysis of the baseline factors associated with the selection of the neoadjuvant chemotherapy regimen

Factors	All subjects, No.	Anthracycline‐based chemotherapy, No. (%)	Anthracycline‐free chemotherapy, No. (%)	*P*‐value
Patients (*n*)	616	553 (89.8)	63 (10.2)	
Age at diagnosis (years)				0.891
<40	163	146 (89.6)	17 (10.4)	
40–65	430	387 (90.0)	43 (10.0)	
≥65	23	20 (87.0)	3 (13.0)	
Menopausal status				0.215
Premenopausal	307	280 (91.2)	27 (8.8)	
Postmenopausal	268	236 (88.1)	32 (11.9)	
Unknown	41	37	4	
BMI				0.677
Normal (<24)	288	256 (88.9)	32 (11.1)	
Overweight (≥24)	279	251 (90.0)	28 (10.0)	
Unknown	49	46	3	
Comorbidity				0.695
Yes	102	91 (89.2)	11 (10.8)	
No	473	428 (90.5)	45 (9.5)	
Unknown	41	34	7	
Family history of breast cancer				>0.900
Yes	23	21 (91.3)	2 (8.7)	
No	567	509 (89.8)	58 (10.2)	
Unknown	26	23	3	
Tumor grade at diagnosis (IDC)				0.056
I+II	85	71(83.5)	14(16.5)	
III	43	41(95.3)	2(4.7)	
NA or Unknown	488	441	47	
Tumor size stage at diagnosis				**0.021**
1	57	46 (80.7)	11 (19.3)	
2	191	169 (88.5)	22 (11.5)	
3	93	89 (95.7)	4 (4.3)	
4	81	75 (92.6)	6 (7.4)	
Unknown	194	174	20	
Lymph node stage at diagnosis				0.690
0	44	37 (84.1)	7 (15.9)	
1	72	64 (88.9)	8 (11.1)	
2	38	35 (92.1)	3 (7.9)	
3	22	20 (90.9)	2 (9.1)	
Unknown	440	397	43	
Tumor stage at diagnosis				**0.009**
I	14	11 (78.6)	3 (21.4)	
II	118	110 (93.2)	8 (6.8)	
III	178	168 (94.4)	10 (5.6)	
IV	35	28 (80.0)	7 (20.0)	
Unknown	271	236	35	
Histological subtype				0.056
IDC	455	415 (91.2)	40 (8.8)	
Non‐IDC	131	112 (85.5)	19 (14.5)	
NA or unknown	30	26	4	
ER status				0.680
Positive	298	267 (89.6)	31 (10.4)	
Negative	267	242 (90.6)	25 (9.4)	
Unknown	51	44	7	
PR status				0.754
Positive	248	222 (89.5)	26 (10.5)	
Negative	320	289 (90.3)	31 (9.7)	
Unknown	48	42	6	
HER2 status				0.838
Positive	240	218 (90.8)	22 (9.2)	
Negative	289	261 (90.3)	28 (9.7)	
Unknown	87	74	13	
Ki−67 at diagnosis (%)				0.200
<40	122	112 (91.8)	10 (8.2)	
≥40	104	90 (86.5)	14 (13.5)	
Unknown	390	351	39	

Significant *p*‐values are indicated in bold.

Abbreviations: BMI, body mass index; ER, estrogen receptor; HER2, human epidermal growth factor receptor 2; IDC, invasive ductal carcinoma; NA, not applicable; PR, progesterone receptor.

### 3.4 Clinicopathological factors associated with the selection of ABC as adjuvant chemotherapy

A total of 2651 patients were subjected to adjuvant chemotherapy. Among these patients, 2165 (81.7%) were treated with ABC, and 486 (18.3%) with AFC (Table 3). ABC was more often used in patients of young age (82.6% for <40 years, *p* < 0.001), those without comorbidity (82.7%, *p =* 0.047), those with low tumor size stage (84.6% for stage 1 + 2, *p =* 0.001), high lymph node involvement (84.9% for stage 2 + 3, *p =* 0.001), IDC (83.2%, *p* < 0.001), or subjected to mastectomy (82.4%, *p =* 0.012) compared with counterparts (Table 3). The remaining factors, such as menopausal status, pTNM stage, and molecular subtypes, had no significant effect on the selection of adjuvant treatment regimen.

**TABLE 3:** Analysis of the clinicopathological factors associated with the selection of the adjuvant chemotherapy regimen

Factors	All subjects, No.	Anthracycline‐based chemotherapy, No. (%)	Anthracycline‐free chemotherapy, No. (%)	*p*‐value
Patients (*n*)	2651	2165 (81.7)	486 (18.3)	
Age at diagnosis (years)				**<0.001**
<40	777	642 (82.6)	135 (17.4)	
40–65	1804	1482 (82.2)	322 (17.8)	
≥65	70	41 (58.6)	29 (41.4)	
Menopausal status				0.094
Premenopausal	1240	1028 (82.9)	212 (17.1)	
Postmenopausal	1207	969 (80.3)	238 (19.7)	
Unknown	204	168	36	
BMI				0.254
Normal (<24)	1262	1043 (82.6)	219 (17.4)	
Overweight (≥24)	1090	881 (80.8)	209 (19.2)	
Unknown	299	241	58	
Comorbidity				**0.047**
Yes	481	379 (78.8)	102 (21.2)	
No	2032	1680 (82.7)	352 (17.3)	
Unknown	138	106	32	
Family history of breast cancer				0.162
Yes	120	104 (86.7)	16 (13.3)	
No	2384	1946 (81.6)	438 (18.4)	
Unknown	147	115	32	
Tumor grade (IDC)				0.581
I+II	862	732 (84.9)	130 (15.1)	
III	481	403 (83.8)	78 (16.2)	
NA or Unknown	1308	1030	278	
Tumor size stage				**0.001**
1+2	1622	1373 (84.6)	249 (15.4)	
3+4	267	204 (76.4)	63 (23.6)	
Unknown	762	588	174	
Lymph node stage				**0.001**
0+1	1623	1292 (79.6)	331 (20.4)	
2+3	1028	873 (84.9)	155 (15.1)	
pTNM stage				0.265
I	210	165 (78.6)	45 (21.4)	
II	833	697 (83.7)	136 (16.3)	
III	921	775 (84.1)	146 (15.9)	
IV	81	68 (84.0)	13 (16.0)	
Unknown	606	460	146	
Breast surgery				**0.012**
Lumpectomy	211	159 (75.4)	52 (24.6)	
Mastectomy	2295	1890 (82.4)	405 (17.6)	
Other/Unknown	145	116	29	
Lymph node examination				0.677
SLNB	31	24 (77.4)	7 (22.6)	
ALND	1928	1614 (83.7)	314 (16.3)	
SLNB+ALND	60	53 (88.3)	7 (11.7)	
No surgery	90	76 (84.4)	14 (15.6)	
Other	8	6 (75.0)	2 (25.0)	
Unknown	534	392	142	
Histological subtype				**<0.001**
IDC	2149	1789 (83.2)	360 (16.8)	
non‐IDC	397	300 (75.6)	97 (24.4)	
Unknown	105	76	29	
ER status				0.720
Positive	1267	1057 (83.4)	210 (16.6)	
Negative	974	807 (82.9)	167 (17.1)	
Unknown	410	301	109	
PR status				0.326
Positive	1153	968 (84.0)	185 (16.0)	
Negative	1097	904 (82.4)	193 (17.6)	
Unknown	401	293	108	
HER2 status				0.580
Positive	810	677 (83.6)	133 (16.4)	
Negative	1321	1116 (84.5)	205 (15.5)	
Unknown	520	372	148	
Ki−67 (%)				0.149
<40	707	603 (85.3)	104 (14.7)	
≥40	435	357 (82.1)	78 (17.9)	
Unknown	1509	1205	304	

Significant *p*‐values are indicated in bold.

Abbreviations: ALND, axillary lymph node dissection; BMI, body mass index; ER, estrogen receptor; HER2, human epidermal growth factor receptor 2; IDC, invasive ductal carcinoma; NA, not applicable; PR, progesterone receptor; pTNM, pathology TNM; SLNB, sentinel lymph node biopsy.

### 3.5 Clinicopathological factors associated with the selection of ABC as advanced first‐line chemotherapy

As regard the first‐line palliative chemotherapy, 713 (23.1%) patients were treated with ABC, while 2380 (76.9%) with AFC (Table 4). Among patients receiving ABC, anthracycline‐containing protocols were more frequently chosen for patients with pTNM stage IV (61.7%, *p* < 0.001), no breast surgery (52.4%, *p* < 0.001), non‐IDC (29.6%, *p* < 0.001), estrogen receptor (ER) positive status (24.6%, *p* < 0.001), DFS ≤2 years (27.4%, *p* < 0.001), or no previous use of anthracyclines (36.9%, *p* < 0.001). Additionally, the statistical results revealed a substantial link between early lymph node involvement (stage 0, 27.0%, *p =* 0.001), no lymph node examination (27.6%, *p =* 0.013), progesterone receptor (PR) positive status (23.5%, *p =* 0.004), no visceral involvement at first recurrence (25.5%, *p =* 0.004), or local treatment (25.0%, *p =* 0.030) and more choice of ABC. No other significant differences were observed on the basis of pre‐defined clinicopathological factors.

**TABLE 4:** Analysis of the clinicopathological factors associated with the selection of the advanced first‐line chemotherapy regimen

Factors	All subjects, No.	Anthracycline‐based chemotherapy, No. (%)	Anthracycline‐free chemotherapy, No. (%)	*p*‐value
Patients (*n*)	3093	713 (23.1)	2380 (76.9)	
Age at diagnosis (years)				0.131
<40	874	183 (20.9)	691 (79.1)	
40–65	2119	502 (23.7)	1617 (76.3)	
≥65	100	28 (28.0)	72 (72.0)	
Menopausal status				0.774
Premenopausal	1451	333 (22.9)	1118 (77.1)	
Postmenopausal	1423	333 (23.4)	1090 (76.6)	
Unknown	219	47	172	
BMI				0.611
Normal (<24)	1493	355 (23.8)	1138 (76.2)	
Overweight (≥24)	1272	292 (23.0)	980 (77.0)	
Unknown	328	66	262	
Comorbidity				0.672
Yes	587	140 (23.9)	447 (76.1)	
No	2354	542 (23.0)	1812 (77.0)	
Unknown	152	31	121	
Family history of breast cancer				0.704
Yes	140	30 (21.4)	110 (78.6)	
No	2784	635 (22.8)	2149 (77.2)	
Unknown	169	48	121	
Tumor grade (IDC)				0.284
I+II	893	139 (15.6)	754 (84.4)	
III	524	93 (17.7)	431 (82.3)	
NA or Unknown	1676	481	1195	
Tumor size stage				0.101
1+2	1729	310 (17.9)	1419 (82.1)	
3+4	326	71 (21.8)	255 (78.2)	
Unknown	1038	332	706	
Lymph node stage				**0.001**
0	1209	326 (27.0)	883 (73.0)	
1	752	150 (19.9)	602 (80.1)	
2	624	133 (21.3)	491 (78.7)	
3	508	104 (20.5)	404 (79.5)	
pTNM stage				**<0.001**
I	231	34 (14.7)	197 (85.3)	
II	865	146 (16.9)	719 (83.1)	
III	962	150 (15.6)	812 (84.4)	
IV	235	145 (61.7)	90 (38.3)	
Unknown	800	238	562	
Breast surgery				**<0.001**
Mastectomy	2492	490 (19.7)	2002 (80.3)	
Lumpectomy	247	59 (23.9)	188 (76.1)	
No surgery	267	140 (52.4)	127 (47.6)	
Other	58	16 (27.6)	42 (72.4)	
Unknown	29	8	21	
Lymph node examination				**0.013**
SLNB	38	8 (21.1)	30 (78.9)	
ALND	2085	433 (20.8)	1652 (79.2)	
SLNB+ALND	60	3 (5.0)	57 (95.0)	
No surgery	116	32 (27.6)	84 (72.4)	
Other	8	1 (12.5)	7 (87.5)	
Unknown	786	236	550	
Histological subtype				**<0.001**
IDC	2320	444 (19.1)	1876 (80.9)	
Non‐IDC	517	153 (29.6)	364 (70.4)	
Unknown	256	116	140	
ER status				**<0.001**
Positive	1494	367 (24.6)	1127 (75.4)	
Negative	1095	191 (17.4)	904 (82.6)	
Unknown	504	155	349	
PR status				**0.004**
Positive	1341	315 (23.5)	1026 (76.5)	
Negative	1253	236 (18.8)	1017 (81.2)	
Unknown	499	162	337	
HER2 status				0.755
Positive	919	187 (20.3)	732 (79.7)	
Negative	1504	314 (20.9)	1190 (79.1)	
Unknown	670	212	458	
Ki−67 (%)				
<40	746	129 (17.3)	617 (82.7)	
≥40	461	72 (15.6)	389 (84.4)	
Unknown	1886	512	1374	
DFS				**<0.001**
≤2 years	1557	427 (27.4)	1130 (72.6)	
>2 years	1536	286 (18.6)	1250 (81.4)	
Metastatic sites				0.527
Distant	2021	452 (22.4)	1569 (77.6)	
Local	521	125 (24.0)	396 (76.0)	
Both	471	115 (24.4)	356 (75.6)	
Unknown	80	21	59	
Visceral involvement at first recurrence				**0.004**
Yes	1558	328 (21.1)	1230 (78.9)	
No	1497	381 (25.5)	1116 (74.5)	
Unknown	38	4	34	
Previous use of anthracyclines				**<0.001**
Yes	2135	251 (11.8)	1884 (88.2)	
No	344	127 (36.9)	217 (63.1)	
Unknown	614	335	279	
Local treatment				**0.030**
Yes	1209	302 (25.0)	907 (75.0)	
No	1780	384 (21.6)	1396 (78.4)	
Unknown	104	27	77	

Significant *p*‐values are indicated in bold.

Abbreviations: ALND, axillary lymph node dissection; BMI, body mass index; DFS, disease‐free survival; ER, estrogen receptor; HER2, human epidermal growth factor receptor 2; IDC, invasive ductal carcinoma; NA, not applicable; PR, progesterone receptor; pTNM, pathology TNM; SLNB, sentinel lymph node biopsy.

### 3.6 Initial recurrence after adjuvant chemotherapy

The initial recurrence of patients receiving adjuvant chemotherapy is summarized in Figure S1. A total of 2651 patients showed tumor recurrence after follow‐up, including 468 local recurrences, 1760 distant metastases, and 357 local plus distant recurrence. Among them, a significant correlation was found between patients who experienced distant metastasis and ABC treatment (69.7%, *p =* 0.001). With regard to the visceral involvement, the proportion of patients with or without visceral involvement was similar when they were treated with ABC (49.9% vs. 50.1%). Among the patients who received ABC, the most common site of the first recurrence was the bone (38.5%), followed by the lungs (31.1%) and liver (22.4%).

## 4 DISCUSSION

In the present study, our findings demonstrated that ABC was at the forefront of breast cancer chemotherapy. In the overall cohort, the vast majority of breast cancer patients received anthracyclines as a chemotherapeutic agent, especially in neo‐ and adjuvant therapy, where nearly 90% and 80% of patients were treated with anthracyclines, respectively. The most commonly used treatment options for (neo)adjuvant settings were anthracycline‐taxane based regimens, as well as taxanes, oral fluorouracils, and taxanes for palliative first to third‐line regimens, respectively.

ABC is usually selected in patients with a high risk of recurrence due to the cumulative dose‐dependent cardiotoxicity.^12^, ^13^ Epidemiological data involving 1116 patients from the University of California indicated that the addition of anthracyclines to chemotherapeutic protocols declined from 95% in 2000–2005 to 65% during the following five years.^14^ A population study in China also revealed that the use of ABC without taxanes was included in 55% of the chemotherapeutic regimens in 2003 compared with 25% in 2008.^15^ Although a growing body of research showed a declining use of ABC, it is still widely prescribed. The results of a previous pooled analysis of four observational studies demonstrated that 61.9% of the early‐stage breast cancer were treated with ABC together with docetaxel, and our results are consistent with these ones.^16^

According to our results and previous studies, more patients with breast cancer in China received ABC than patients in western countries.^13^, ^17^ Moreover, with the increase of the treatment line, the proportion of patients receiving monotherapy increased, along with the use of oral chemotherapeutic agents such as CAP/S‐1. Anthracyclines showed the same trend, but the combination regimen was still dominant. A potential explanation might be that anthracyclines were covered by medical insurance in China, and a limited supply of novel therapeutic regimens was available at that time. In addition, Chinese patients pay more attention to the efficacy of the therapy and have a higher tolerance for the adverse reactions caused by the treatment compared with breast cancer patients in western countries, promoting the application of cytotoxic drugs and combination therapy in China. This feature in Chinese breast cancer patients was also highlighted in other studies concerning treatment approaches.^18^

As suggested by our study, the selection of ABC was markedly associated with several clinicopathological factors. The overall body of evidence showed that patients who were young, in a premenopausal stage, without comorbidity, and with a severe disease (IDC, lymph node involvement, high pTNM stage, subjected to mastectomy or SLNB+ALND, and shorter DFS) represented the categories of patients more likely treated with ABC. When the neoadjuvant regimen was used, a significant association was found between patients treated with anthracyclines and high malignancy (such as large tumor size, cTNM stage III disease, IDC, or grade III tumor), which was similar to the association observed in the entire cohorts. However, in the adjuvant setting, the patients treated with ABC were more often young, with no comorbidity, with a small tumor size together with high lymph node stages, and subjected to mastectomy. Anthracyclines are cytotoxic antineoplastic drugs with a potent activity against breast carcinoma and are one of the preferred agents selected for breast cancer therapy at all stages. In the (neo‐) adjuvant settings, ABC is more likely to be selected for patients with higher TNM stages or pathological grades of early breast cancer. Moreover, anthracyclines require patients to tolerate the adverse reactions of medications, and young patients with better organ function may have more chance to receive ABC. When considering ABC as the advanced frontline therapy, more potential factors seem to be involved. Among patients using ABC, some might be newly diagnosed with advanced breast cancer; some might have favorable pathological stages or subtypes after radical surgery, and anthracyclines were chosen to combat the recurrence or metastasis rather than as adjuvant therapy. In addition, the previous absence of ABC increased the chance in choosing ABC during the palliative setting.

Emerging studies emphasized the adverse effects of anthracyclines, and further efforts have been made to prevent and treat them, especially against cardiotoxicity.^19^, ^20^, ^21^ Several drugs and/or therapies are proposed as cardioprotective agents/approaches during the treatment with ABC, such as dexrazoxane, β‐blockers, ACE inhibitors, telomerase therapy, and matrix metalloproteinase inhibitors.^22^, ^23^, ^24^, ^25^, ^26^ However, it is also critical to determine the factors influencing the selection of ABC. Unfortunately, few studies addressed this question, and more investigations are needed. In a study on hormone receptor‐positive early‐stage breast cancer, ABC was administered more often to young patients (40% of the patients <65 years), to the ones with stage III disease (69%) or higher 21‐gene recurrence scores, and positive lymph nodes encouraged the selection of ABC in the absence of high recurrence scores.^9^ As regard the clinicopathological characteristics, the results of this study were highly compatible with that of our analysis. Additionally, our analysis revealed that ABC administration was correlated with distant metastasis, which might be due to its use in patients with severe diseases, together with the resistance to anthracyclines.^27^

Several limitations in this work should be mentioned. Above all, its retrospective nature study is associated with limitations, including temporal lag, missing data, a broad spectrum of treatment regimens, and slight differences in the treatment of patients in each hospital, making difficult to control all potential confounders. The retrospective nature also precludes the attribution of a link between clinicopathological features and the selection of anthracyclines, supporting the need of further studies on this topic. Furthermore, our study is underpowered to assess the efficacy of chemotherapy and clinical outcome due to the observational nature and insufficient medical records available. Besides, a slight bias was present in the analysis of neo‐ and adjuvant chemotherapy, since the participants enrolled in this study were all patients with advanced breast cancer. However, despite the above‐mentioned limitations, the present study also possesses strengths, such as a large and representative sample size, reliable data, and rigorous analyses; thus, it would be useful to optimize the clinical use of ABC.

Collectively, ABC was still used as the main component of the chemotherapeutic regimens to combat breast cancer, and it was the most frequently used protocol in the neo‐ and adjuvant settings. Combination therapy was the predominant mode of ABC administration, although at the palliative stages, with the increase of the number of the treatment lines, the proportion in the use of monotherapy expanded. The correlation between clinicopathological characteristics and the choice of ABC varied at different settings, suggesting the preference and different perspectives of medication considered by medical oncologists in the use ABC in China. To date, several promising predictors of ABC efficacy or resistance in breast cancer have been proposed and assessed, such as GR, NUP98, FKBP12, and ERCC,^28^, ^29^, ^30^, ^31^ to guide the selection of the appropriate treatment and maximize the benefit of chemotherapy for patients, avoiding unnecessary adverse events, costs, and risk of progression.

## CONFLICT OF INTEREST

The authors declare that there are no conflicts of interest.

## ETHICS STATEMENT

This work was approved by the Ethics Committee of National Cancer Center/National Clinical Research Center for Cancer/Cancer Hospital, Chinese Academy of Medical Sciences and Peking Union Medical College (Ref. 15‐115/1042). Patient consent for this study was not required as there were no risks anticipated to the enrolled participants. All data analyzed were in aggregate information and were stripped of any patient identifiers.

## Supporting information

Fig S1

## Data Availability

The original contributions presented in the study are included in this publication. Further inquiries can be directed to the corresponding authors.
