# Supplementary figures and images for: Profile, treatment patterns, and influencing factors of anthracycline use in breast cancer patients in China: A nation‐wide multicenter study
Source: Cancer Med. 2021 Sep 2;10(19):6744–61. doi: 10.1002/cam4.4215 (PMC8495288; doi:10.1002/cam4.4215)

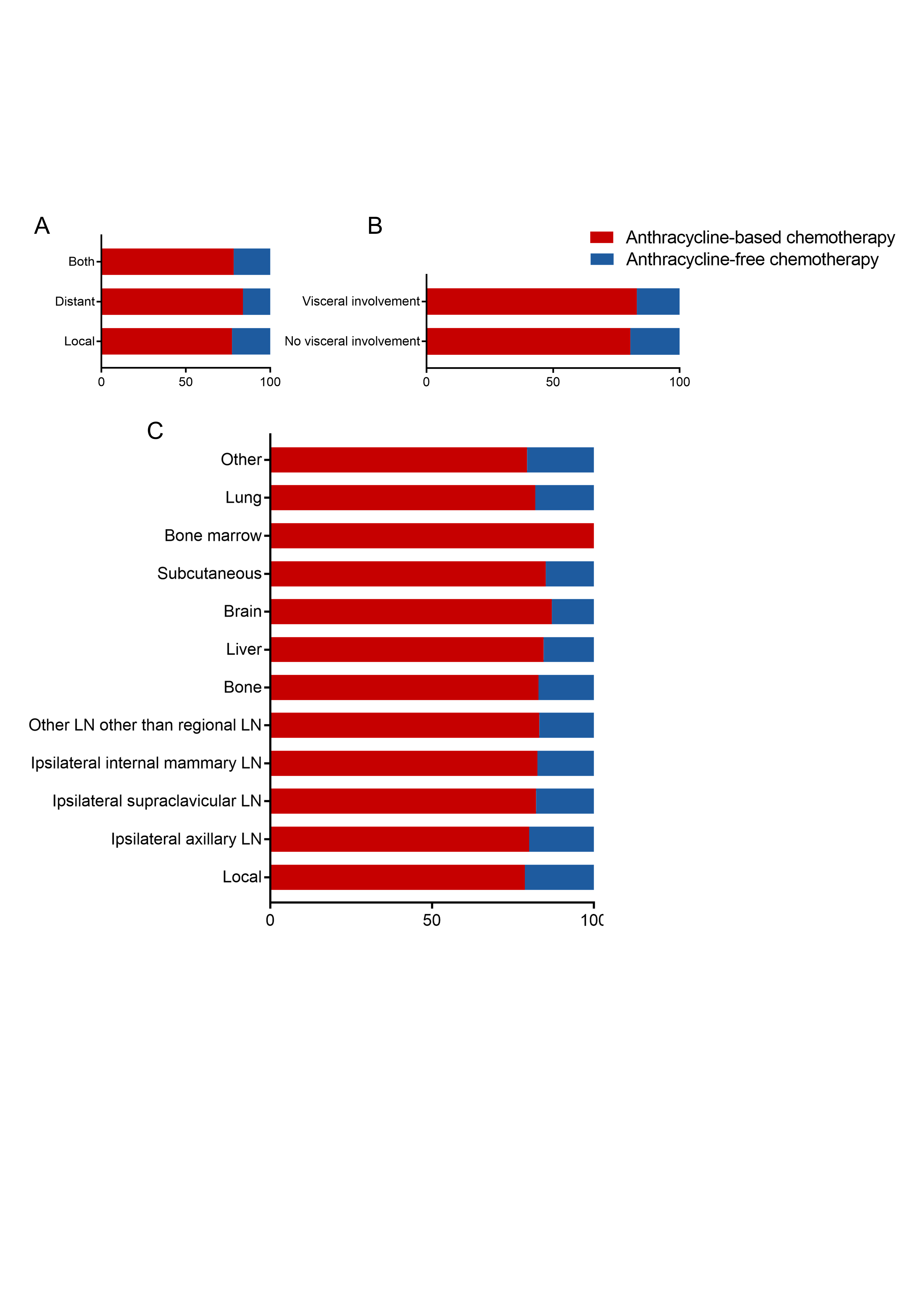

Supplement: Supplementary file 1 — Fig S1 [file CAM4-10-6744-s001.tif]
